# Neonatal mortality and causes of death in Kersa Health and Demographic Surveillance System (Kersa HDSS), Ethiopia, 2008–2013

**DOI:** 10.1186/s40748-016-0035-8

**Published:** 2016-07-19

**Authors:** Nega Assefa, Yihune Lakew, Betelhem Belay, Haji Kedir, Desalew Zelalem, Negga Baraki, Melake Damena, Lemessa Oljira, Wondimye Ashenafi, Melkamu Dedefo

**Affiliations:** College of Health and Medical Sciences, Haramaya University, P.O.Box 1494, Harar, Ethiopia; Ethiopian Public Health Association, Addis Ababa, Ethiopia; College of Computing and Informatics, Haramaya University, Harar, Ethiopia

**Keywords:** Neonatal deaths, Causes of death, Verbal autopsy, Kersa HDSS, Ethiopia

## Abstract

**Background:**

In the world, Neonatal mortality accounts for 40 % of death of children under the age of 5 years. Majority of neonatal deaths occur in developing countries outside of formal health system, among which death in the first hour of first day of their life constitute the huge bulk. This analysis is intended to estimate neonatal mortality rates and identify the leading causes of death based on the surveillance data over 6 years period in Kersa health and demographic surveillance system (Kersa HDSS) site, Eastern Ethiopia.

**Methods:**

Kersa HDSS is an open dynamic cohort of population established in 2007. The surveillance started after conducting a baseline census followed by population update and events registration on house-to-house visits every 6 months. Data were collected using verbal autopsy (VA) questionnaire from close relatives (usually mothers in this case) and causes of deaths were assigned by 2 to 3 physicians. This analysis was done based on 301 neonatal deaths and 10,934 live births occurred during 2008 to 2013.

**Results:**

The overall neonatal death rate during the study period was 27.5 per 1000 live births. Nearly all neonatal deaths (94 %) occurred at home. More than four-fifth (82.4 %) of the deaths was occurred in the first week of life. More than 80 % of the deaths were due to perinatal causes. Bacterial sepsis of the newborn accounted for 31.2 % followed by birth asphyxia and perinatal respiratory disorder (28.2 %), and prematurity (17.3 %). Higher number of death was observed in Tolla and Bereka sub-districts located at the southern parts of the study site which are away from the main road network.

**Conclusion:**

The overall neonatal mortality over 6 years is the same to the national average (27 per 1000 live births). The leading causes of neonatal death were bacterial sepsis of newborn and birth asphyxia. Community-based skilled health care delivery during birth should be emphasized.

## Background

Under 5 mortality remains 1 of the biggest public health concern constituting 7 · 2 million in 2011, of which 2 · 2 million were early neonatal, and 0 · 7 million late neonatal deaths [1]. Neonatal deaths account for 40 % of under 5 mortality [2]. Based on data collected from 193 countries in 2012, the global and Sub-Saharan neonatal mortality rate was 21 and 32 per 1000 live births, respectively. About 99 % of neonatal deaths were from low and middle income countries, of which 66 % (1.16 million deaths) are in Africa and Southeast Asia [3].

In the same report, the top 5 countries with the greatest number of neonatal deaths were India, Nigeria, Pakistan, China, and Democratic Republic of Congo. According to the 2013 and 2014 report, Ethiopia ranked 6^th^ in neonatal deaths [3, 4]. The 2012-2014 global data indicated that the 3 major causes of neonatal death were preterm birth complications (35 %), neonatal infections (23 %) and intrapartum related complications (23 %). These causes account for nearly 80 % of deaths in this age group [3–8].

Majority of the neonatal deaths in developing countries occur outside the formal health system. As a result, many studies on the causes of death of children under the age of 5 excluded neonatal deaths mainly for 2 reasons; 1 is poor reporting on a sign and symptoms that leads to death and the other is the number of cases reported is very small in number. To resolve this, some researchers tried to group neonatal deaths as ‘other’ childhood or ‘perinatal’ causes, which has created difficulty to identify causes deaths associated with the neonatal [9]. Recently, several studies have focused exclusively on the use of VA for the newborn deaths, making this an opportune time to assess the current state of knowledge and provide direction for future research efforts [10].

In Ethiopia, due to the absence of vital event registration, most deaths are left undocumented. As the vast majority of deaths occur outside the health system, the ability of health facilities to generate representative statistics is limited in the country [9]. In this regard, the use of verbal autopsy (VA) from health and demographic surveillance system is the most promising interim solution for this problem [11–13].

Reducing under 5 mortality rate by two third was Millennium Development Goal number 5 (MDG-5) that ends in 2015 and Ethiopia is 1 of the countries achieved this goal. However, the rates disaggregated by age showed that neonatal mortality continues to be suspended high with little or no sign of reduction over years [5, 14]. Focusing on under-five mortality in general and neonatal mortality in particular continues to be the focus during Sustainable Development Goal (SDG) period [15].

Consistent to the SDG goals, Ethiopia has also developed a health sector strategic plan to direct its health intervention through 2035. In this plan, the country aspires to be a middle income country averting unnecessary neonatal mortality [16]. Therefore, it is imperative to substantiate with findings on the ground if the country is in the right direction over the years bench marking the end period of MDG.

Hence, information generated using the existing data from Kersa HDSS will help in providing estimate of neonatal mortality and the leading causes of death to support evidence-based decision making at different levels.

## Methods

### Kersa HDSS Site

**Fig. 1:**
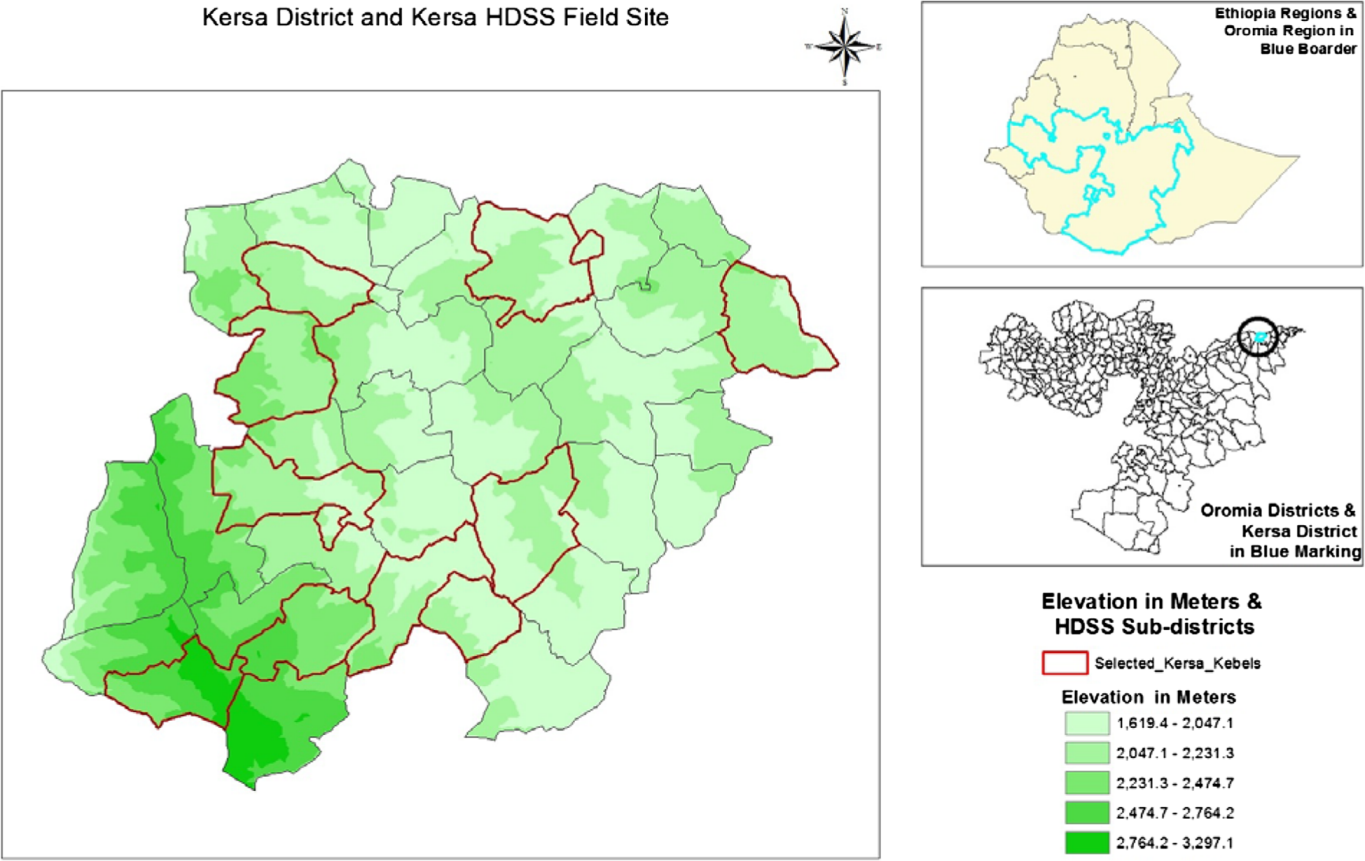
Kersa District and Kersa HDSS, Ethiopia

The site was established in September 2007 and located at the eastern Hararge of the Oromyia regional state in eastern Ethiopia. The district capital, Kersa, is located 44 km from Harar, west direction. The district has 3 climatic zones with the altitude ranging from 1400 to 3200 meters above sea level (Fig. 1). This surveillance site covers 12 kebeles, the lowest administrative units (2 urban and 10 rural) from 38 randomly selected kebeles (As of January 2015, it is increased to 24 Kebeles). The site started its surveillance on 10,256 households and 52,470 population in 2007 and by 2013, the population has increased to 62,550. In the move to double the size of population and households under surveillance, by 2015, as the number of Kebeles doubled from 12 to 24, the size of the population has increased to 129,000 and the number of households to 27,000. Kersa HDSS is a member of INDEPTH network of Health and Demographic Surveillance System (HDSS) sites in the world [17]. In Kersa HDSS, there are a total of 19 health facilities; including 4 health centers, 10 health posts and 5 clinics. There are 7 research team members, 22 vital events enumerators, 4 verbal autopsy interviewers (VAIs), 5 field supervisors, 2 data managers, and 6 data clerks [18].

### Study design and population

Kersa HDSS is an open dynamic cohort study design that longitudinally follow a well-defined entities or primary subjects (individuals, households, and residential units) and all related socio-demographic and health related outcomes within a clearly defined geographic area. The surveillance was started by conducting a baseline census of the population including housing and socio-economic characteristics. Then, population update and events registration on house-to-house visits to register all pregnancy observations and outcomes, deaths, marriage and in-and-out migration has been done every 6 months. Subsequently, any causes of death in the population from registered deaths were identified using Verbal Autopsy (VA) [18].

### Verbal autopsy questionnaire

There are 3 verbal autopsy forms (deaths up to 28 completed days of life-neonatal deaths; deaths to children between 4 weeks and 14 years of age; and deaths to persons aged 15 years and above), adopted from the 2007 standardized World Health Organization (WHO) questionnaires [19–21]. The verbal autopsy forms and questionnaire was translated in to Oromefa language for use in the field. The questionnaires were used for collecting information, such as, age, sex, place of death, sign and symptoms observed during the late life period of the deceased. In addition, a short narrative history of the course of disease that leads to death, health services used in the period before death, and documentation of any medical evidence available at the household, including whether a health worker informed the respondent of the cause of death. Particularly, for the neonatal form, the condition of the mother during and after pregnancy and birth was used. The questionnaire also contain the symptom duration checklist arranged loosely around anatomical systems and are intended to be as informative as possible in leading a positive diagnosis of probable cause of death, as well as, the confident exclusion of differential diagnoses [18].

### VA data collection procedure

The process begins with a report of a death occurring to a resident of the area by the local data collectors. The regular vital event interviewers receive these reports and maintain records of deaths that occur in their working areas using event recording form. The information in the death event recording form is later transferred to death registration book that is kept in the field office for referral purpose. Copy of the recording form with location information is given to the VA interviewer to complete the VA interview after the appropriate mourning period. The vital event interviewers help the VA interviewers to arrange an appointment with the family of the deceased to conduct the VA interview. This was within 1-3 months after a death with due consideration to culturally appropriate mourning periods. On the day of interview, the VA interviewer arrives at the residence of the deceased to conduct the interview with an adult person (with a mother in this case) at the deceased households. When it is difficult to get a reliable respondent, the VA interviewer arranges an appointment to visit the household on another day when a more informed respondent will be available. Up to 3 attempts were made to conduct an interview if the information given is incomplete after 3 visits, the VA interviewer completes the VA form with the information that is available. A note that the interview is incomplete due to the absence of reliable respondents was made on the form in the ‘history of events’ section. These events are counted as deaths in the system, although the cause of death will remain unknown. Again, every section of the form was filled in accurately before the form is submitted to the research team for onward processing.

### Causes of death assignment

Two physicians, trained in VA diagnosis and coding procedures for the study, assigned codes and titles, up to 3 causes of death (underlying, immediate and contributing factor) independently using information contained in VA forms based on the WHO International Classification of Disease-10 (ICD-10) and VA code system. After checking agreements of physician assigned underlying cause of death based on VA coding discordant cases were sent to the third physician again for independent review and diagnosis. If any of 2 of these 3 physicians assigned an underlying cause of death to the same VA code, this was considered as the final cause of death; otherwise, the causes were labeled as undetermined. In the analysis, 18 cases (6 %) cause of death were not determined based on the 3 physician report.

### VA data quality controls

Individuals who have completed at least a high school education and who have been working in the field during the last 1 year was continued to work as VA interviewers. They received 3 days training on the questionnaires, recording, contacting close relatives and data collection procedures. The training curriculum include sessions on discussion of individual symptoms, and their description in local language for easy recognition by the respondents and demonstration of interviewing techniques by research team members. The VA interviewer was informed about new deaths by the resident enumerators and conduct verbal autopsy interviews. Researcher team members coordinate the field activities of all vital events registration and VA interviewers are responsible for making sure that the field operations run smoothly and efficiently and also give supervisory support to events data collectors and VA interviewers. During the course of the fieldwork, supervisors continually visit the sites to check on the progress and sort out problems that may have been encountered by enumerators.

### Data management and analysis

Neonatal mortality rate was calculated using the total neonatal death divided by total number of births during the 6 years period. Causes of death were analyzed by some basic socio-demographic characteristics. Tables and graphs were used to summarize and present the data. STATA version 11 software and excel sheet were used for data analysis. Geographic Information System (GIS) mapping is used to plot the extent of death over the map against the sub-district cumulative deaths. Cross tabulations of death events by sex, age and other background characteristics was provided. 95 % confidence interval for neonatal death rate is calculated for sub districts and *p*-value for 0.05 is taken to determine level of significance.

### Ethical approval

Kersa HDSS site has received ethical clearance from the Ethiopian Science and Technology Agency, Ethiopian Public Health Association (EPHA), US Center for Disease Control and Prevention (CDC) and the Health Research Ethics Review Committee (HRERC) of Haramaya University. To capture occurrence of vital events to any family member, head of a family or an eligible adult among the family was interviewed. Therefore, informed verbal consent was obtained from head of the family or eligible adult among the family. This consent procedure was stated in the proposal which was approved by the ethical review committee. To keep confidentiality, data containing personal identifiers of subjects were not shared to third party.

## Results

During the period from 2008 to 2013, a total of 301 neonatal deaths were recorded. The lowest proportions of neonatal deaths were observed in 2012 (13.0 %) and in 2013 (13.0 %). The highest proportion of neonatal deaths were seen in 2009 (20.9 %) and in 2010 (20.6 %) (Table 1). As shown in Table 2, the overall neonatal death rate in the study period was 27.5 per 1000 live births.

**Table 1:** Demographic characteristics of the deceased by surveillance years, Kersa HDSS site in Ethiopia

Background characteristics	Surveillance years and number of neonatal deaths							
	2008	2009	2010	2011	2012	2013	2008–2013	
	n	n	n	n	n	n	n	Death fraction with 95 % CI
Residence								
Urban	3	3	1	9	0	1	17	5.6 [3.44–8.71]
Rural	44	60	61	42	39	38	284	94.4 [91.29–96.56]
Sex								
Male	28	34	44	32	23	23	184	61.1 [55.53–66.52]
Female	19	29	18	19	16	16	117	38.9 [33.48–44.47]
Age at death								
0–7 days	47	53	52	41	29	26	248	82.4 [77.78–86.39]
8–28 days	0	10	10	10	10	13	53	17.6 [13.61–22.25]
Total	47	63	62	51	39	39	301

**Table 2:** Neonatal death rate over the study period between 2008 and 2013, Kersa HDSS in Ethiopia

Surveillance years	Neonatal deaths	Live births	NMR with 95 % CI
2008	47	1616	29.1 [21.7–38.2]
2009	63	1756	35.9 [27.9–45.4]
2010	62	1983	31.3 [24.3–39.6]
2011	51	1549	32.9 [24.9–42.7]
2012	39	1770	22.0 [15.9–29.7]
2013	39	2260	17.3 [12.5–23.3]
2008–2013	301	10,934	27.5 [24.6–30.7]

NMR-Neonatal Mortality Rate

Over the 6 years period (2008-2013) 61 % of the deceased were male neonates and 39 % were females. Among the deceased neonates, more than four-fifth (82.4 %) of the deaths occurred in the first week of birth and the rest were in the remaining 3 weeks of neonatal period.

**Fig. 2:**
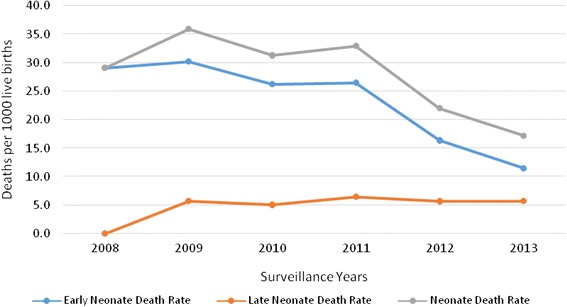
Pattern of Neonatal death rates over years (2008–2013), Kersa HDSS, Ethiopia

Although it shows persistent decline, the pattern of neonatal and early neonatal death rates over the course of the study period was significantly higher than late neonatal death rates. The late neonatal death rate remains relatively constant over the study period with no declining pattern (Table 2 and Fig. 2).

**Fig. 3:**
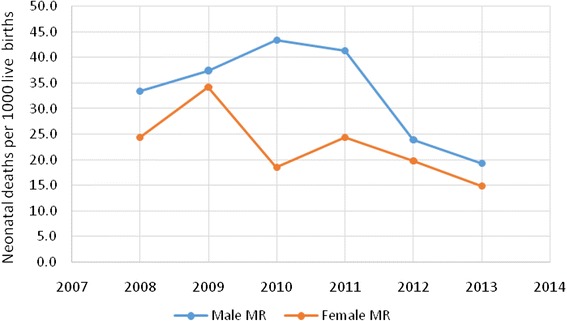
Difference between male and female neonatal death over the years (2008–2013), Kersa HDSS, Ethiopia

Over the 6 years period, a statistically significant higher death rate was observed among male newborns compared to female newborns. The gap in male and female newborn deaths was highest in 2010. A decreasing pattern was also documented in both male and female newborn death rates over the study period (Fig. 3).

**Fig. 4:**
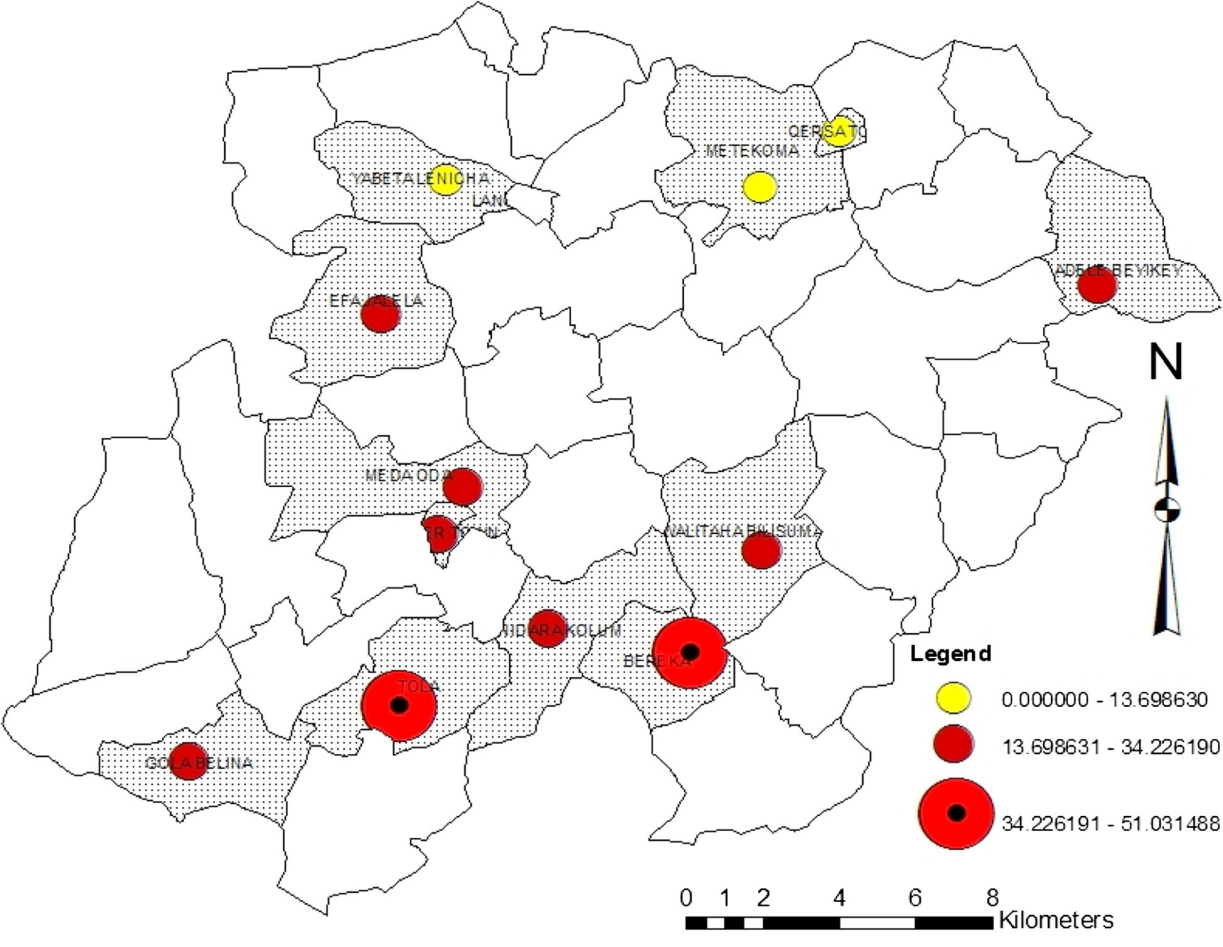
Sum of 2008–2013 Neonatal death rate distribution in sub-districts of Kersa HDSS, Ethiopia

Of the total neonatal deaths observed during the 6 years period, the vast majority (94.4 %) occurred in rural parts of the study area, and only 17 (5.6 %) were occurred in urban areas. Nearly all neonatal deaths (94 %) occurred at home and the remaining were in hospital and health centers. As depicted in Fig. 4, over the study period, a relatively highest neonatal death rate was recorded in 2 villages of Bereka and Tolla followed by other neighboring villages that are located in southern parts of the study site.

### Causes of neonatal deaths

**Fig. 5:**
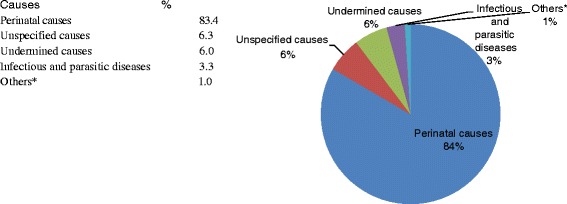
Percent of broad causes of neonatal death in 2008–2013, Kersa HDSS, Ethiopia. *Others include diseases of the circulatory system, external causes of death and gastrointestinal disorders with cases less than 5

Based on the broad classification of causes of neonatal deaths, more than 80 % of the deaths were due to perinatal causes. Deaths due to circulatory diseases, external causes and gastro-intestinal diseases account for only 1 % of neonatal deaths while 3.3 %, 6 %, and 6.3 % of neonatal deaths were due to infectious or parasitic diseases, undetermined causes and unspecified causes, respectively (Fig. 5).

With regard to the specific causes of neonatal death, bacterial sepsis of the newborn accounts for 31.2 %, while it is closely followed by birth asphyxia and perinatal respiratory disorder (28.2 %), and prematurity (17.3 %). Other causes of deaths are other diseases related to the perinatal period (3.3 %) and acute lower respiratory infection (2.7 %) (Table 3).

**Table 3:** Specific neonatal causes of death from 2008–2013, Kersa HDSS in Ethiopia

Causes of death	2008–2013	
	Deaths	Cause of death fraction with 95 % CI
Acute lower respiratory infection	8	2.7 [1.24–4.98]
Bacterial sepsis of newborn	94	31.2 [26.18–36–64]
Birth asphyxia and perinatal respiratory disorder	85	28.2 [23.37–33.53]
Other diseases related to the perinatal period	10	3.3 [1.70–5.84]
Prematurity (including respiratory distress)	52	17.3 [13.32–21.86]
Others ^a^	15	5.0 [2.92–7.91]
Undetermined cause of death	18	6.0 [3.70–9.11]
Unspecified causes of death	19	6.3 [3.96–9.51]
Total	301	

^a^ Birth trauma, congenital malformation, other and unspecified, congenital malformation of nervous system, congestive heart failure, contact with unspecified venomous animal or plant, intestinal infectious diseases including diarrhea, neonatal pneumonia and tetanus neonatorum with cases less than 5

As part of neonatal causes, the leading causes of death for early neonatal period (from birth to 7 days) were birth asphyxia and perinatal respiratory disorder (33.1 %), bacterial sepsis of newborn follows (24.2 %) and prematurity (including respiratory distress) (20.2 %) (Table 4). The late neonatal (8-28 days) causes of deaths where dominated by bacterial sepsis of newborn (64.2 %) and acute lower respiratory infection including pneumonia (7.5 %) (Table 5). As indicated in Table 6, over the study period, in most of the Kebeles, the leading causes of neonatal death were bacterial sepsis of newborn, birth asphyxia and prematurity including respiratory distress.

**Table 4:** Specific early neonatal causes of death from 2008–2013, Kersa HDSS in Ethiopia

Causes of death	2008–2013	
	Deaths	Cause of death fraction with 95 % CI
Bacterial sepsis of newborn	60	24.2 [19.17–29.84]
Birth asphyxia and perinatal respiratory disorder	82	33.1 [27.42–39.10]
Other diseases related to the perinatal period	8	3.2 [1.51–6.03]
Prematurity (including respiratory distress)	50	20.2 [15.52–25.50
Others ^a^	17	6.9 [4.18–10.53]
Undetermined causes of death	15	6.0 [3.56–9.56]
Unspecified causes of death	16	6.5 [3.87–10.05]
Total	248	

^a^ Acute lower respiration including pneumonia, birth trauma, congenital malformation of the nervous system, contact with unspecified venomous animal, intestinal infectious diseases including diarrhea, neonatal pneumonia and tetanus neonatorum

**Table 5:** Specific late neonatal causes of death from 2008–2013, Kersa HDSS in Ethiopia

Causes of death	2008–2013	
	Deaths	Cause of death fraction with 95 % CI
Acute lower respiratory infection including pneumonia	4	7.5
Bacterial sepsis of newborn	34	64.2
Birth asphyxia and perinatal respiratory disorder	3	5.7
Congenital malformation, other and unspecified	1	1.9
Congestive heart failure	1	1.9
Other diseases related to the perinatal period	2	3.8
Prematurity (including respiratory distress)	2	3.8
Undetermined causes of death	3	5.7
Unspecified causes of death	3	5.7
Total	53	100.0

**Table 6:** Neonatal death rates and leading causes per Kebele in the period from 2008–2013, Kersa HDSS site

Study villages	Deaths	LB	NNM rate	Leading causes of death (COD)
Qersa town	5	397	12.6	Bacterial sepsis of newborn
Metekoma	10	730	13.7	Bacterial sepsis of newborn
Yabeta Lencha	8	920	8.7	Bacterial sepsis of newborn
Ifa Jalela	30	1113	27.0	Prematurity including Respiratory Distress (RD)
Mede Oda	23	850	27.1	Bacterial sepsis of newborn and prematurity RD
Weter Town	12	529	22.7	Bacterial sepsis of newborn and birth asphyxia
Handhura Kossum	33	1249	26.4	Bacterial sepsis of newborn and birth asphyxia
Tolla	47	921	51.0	Bacterial sepsis of newborn and birth asphyxia
Gola Belina	23	672	34.2	Bacterial sepsis of newborn and birth asphyxia
Bereka	32	645	49.6	Birth asphyxia
Walteha Bilisuma	29	1378	21.0	Bacterial sepsis of newborn
Adele Key Key	49	1530	32.0	Birth asphyxia

## Discussion

The cumulative average neonatal mortality rate was 27 per 1000 live births. This is lower than many of the reports from countries in Africa and other developing countries in Asia like Pakistan and Bangladesh [3, 4, 22–25]. The rate reported by the present study is consistent to the previous study in south west Ethiopia around Bonke district of Gamo Gofa zone [26], however it is lower than the 35.5 per 1000 live births that has been recently reported from southwest Ethiopia around Jimma Zone [27]. The differences in the reports could be attributed to the timing and differences in the settings of the 2 studies. The lower level of neonatal mortality in the present study could be partly explained due to the fact that the present study was conducted on health and demographic surveillance site (HDSS) and the population in such setting could have better awareness about the health issues. The neonatal mortality rate seems to reduce over the study period, however, the level of cumulative average of neonatal mortality observed in this study is nearly what is considered to be high and calls for policy attention [28].

The findings revealed that neonatal deaths observed over 6 years period were mainly males that nearly accounted for 3 out of 5 neonatal deaths and most deaths occurred at home and among the rural part of the study area. This is in agreement with other reports from elsewhere and in Ethiopia [23, 29]. Consistent to other studies, most of neonatal deaths occurred during the first 7 days of birth [8, 22, 30, 31]. Most neonatal deaths occurred at home and during the first week of life imply that the observed level of neonatal mortality could be attributed to lack of skilled birth assistance as home deliveries are the major place of delivery in the study area [32, 33]. The absence of neonatal Intensive Care Unit (ICU) in the area has also contributed for unabated neonatal mortality.

The study also revealed that sepsis, birth asphyxia and prematurity accounted for three-fourth or more of early neonatal deaths, while sepsis is the commonest cause of late neonatal death, contributing to more than 60 % of neonatal deaths occurring to this age group. These are consistent to the previous reports on the cause of neonatal mortality [4, 5, 8, 24, 26].

Recently, other studies have indicated that sepsis is the major cause of late neonatal death [5]. Delivery complications are associated with odds of neonatal deaths. The world Health organization estimated the most direct causes of neonatal deaths to be preterm birth, severe infection and asphyxia [34].

Previous research from Bangladesh [25], Tanzania [35] Pakistan [36] and Ethiopia [27] have consistently established that birth asphyxia is 1 of the major causes of death. These findings imply that most of the neonatal deaths are mainly due to lack of access to skilled birth assistance and complications of labor and delivery.

The report also indicted the highest number of deaths in the southern part of the district in Tola and Baraka villages. These villages are away from the main road and their access to health care is limited due to terrain and poor feeding road network. This is an important implication to further focus on community based skilled birth attendants in resource limited setting.

Ethiopia has strived to reach every family with basic health service using a home born strategy referred to as ‘Health Extension Program” [37, 38]. The health extension program is designed to provide basic health promotion activities principally to the rural community. Though, the services rendered by this program are sixteen health promotion packages including family planning, antenatal, delivery and postnatal care [39, 40], the toll of neonatal death at the early days of neonatal life persists.

Reports on the rate and cause of neonatal death helps countries to track their performance towards the set national and international targets [15, 16]. Many of the earlier reports are based on estimates of data from clinical setting, which doesn’t represent the health condition in the general population. This report is based on a verbal autopsy for the deceased neonates from the ongoing health and demographic surveillance system in the rural Ethiopia; hence it gives a good bench mark to track the performance of SDG and Ethiopian health sector strategic plan in the future.

The verbal autopsy generate information through conversations with family members of the deceased, the VA method uses information on circumstances leading to death, including symptoms and signs during terminal illness to assign causes of death [41–45]. The VA method has been used in different resource constraint countries to develop a viable mid or long-term strategy for improving neonatal mortality. It is a popular and reliable method for community diagnoses of major causes of death in developing countries, where majority of the deaths occur at home. It has been demonstrated to produce valid estimates of cause-specific mortality fractions in many settings [11].

Limitations of this method are problem of remembering the state of health condition for someone during the late periods of life that introduce recall bias, the reported health problems might not be exactly the same conditions felt by the deceased that introduce reporting error, and the agreement of physician to diagnose based on reported health condition might introduced misclassification. In order to reduce these errors it was advised to collect the information from close relatives with the best knowledge relatives could remember, in this case from the mother or father of the baby [12, 23, 27, 44, 45].

## Conclusion

The cumulative neonatal mortality over 6 year period is 27 per 1000 live birth. Over years it showed a little decline without significant difference between subsequent years. Early neonatal death contributed to majority of the neonatal deaths. Perinatal causes of death; sepsis, birth asphyxia and prematurity accounted for three-fourth or more of early neonatal deaths, while sepsis is the commonest cause of late neonatal death. Increasing access to skilled birth attendant in the community setting through the health extension program by improving the approach to delivery service could avert preventable neonatal deaths.

## Abbreviations

CDC, Center for Disease Control and Prevention; EPHA, Ethiopian Public Health Association; GIS, Geographic Information System; HDSS, Health and Demographic Surveillance System; HRERC, Health Research Ethics Review Committee; ICD-10, International Classification of Disease-10; MDG, Millennium Development Goal; SDG, Sustainable Development Goal; VA, Verbal Autopsy; WHO, World Health Organization
